# Supplementary material for: Antimicrobial effect of oral care gel containing hinokitiol and 4-isopropyl-3-methylphenol against intraoral pathogenic microorganisms
Source: PLoS One. 2023 Sep 1;18(9):e0283295. doi: 10.1371/journal.pone.0283295 (PMC10473516; doi:10.1371/journal.pone.0283295)
Supplement: Attachment — Submitted filename: Response_to_Reviewers.docx [file pone.0283295.s001.docx]

**Response to Reviewers**

We are grateful to the reviewers for their helpful comments that have contributed to the improvement of our research paper. We have provided the responses to the comments below:

**Reviewer #1:** After the reviewing the submitted manuscript, it must be carefully revised as the following:

1-The title must be more attractive and changed to antimicrobial instead of antibacterial.

→Thank you for pointing it out. The title has been changed according to your suggestion.

2-Introduction must be reconstructed to be more informative and contains the problem and how the other publications try to solve this problem and how your paper try to solve that.

→Thank you for your advice. In lines 57–68 and 93–101 of the introduction, we have included the current problem(s) and the solutions from previous studies and our attempts.

3-Deep discussion and related and recent studies must include.

→We have cited a recent research paper in lines 93–101 of the introduction. Moreover, we have included recent studies in the discussion in lines 218–230, 249–251, and 253–270 of the discussion.

4-The novelty and the future perspective in addition to limitation must be included.

→Thank you for your comment. We have revised the discussion and added the novelty and limitations of the research in lines 271–287 and 292–298, respectively.

5-Antibiofilm assay must be included in addition to growth kinetics.

→Thank you for your suggestion. We have added a description of the anti-biofilm assay in the Material and methods section, lines 154–157. In addition, we have added considerations on anti-biofilm assays in lines 253–263 of the discussion.

6-Reaction mechanism determinant must be performed by FESEM, HRTEM, and membrane leakage assay.

→Thank you for your advice. You are absolutely right; we also think that it is necessary to investigate reaction mechanism using FE-SEM and HR-TEM. However, currently there is a limit to what we can do with our current research equipment and funding; therefore, we were unable to perform these experiments. This is a limitation of our research and has been included as a subject for future study in lines 294–298.

7-English Language editing must be performed by English Native Speaker.

→ English language editing has been performed by an English Native Speaker.

**Reviewer #2:** The manuscript entitled “Antibacterial Effect of Oral Care Gel-Containing Hinokitiol and 4-isopropyl-3-methylphenol Against Intraoral Pathogenic Microorganisms” is about to provide the evidence for new anti-intraoral pathogenic microorganism agents.

After first time use, authors can use abbreviation for microorganism’s scientific name. I found *Candida albicans* several times.

→We have corrected the relevant parts in the entire manuscript.

At “Antimicrobial disc diffusion assay”, it is not clear why authors use SCD medium?

Why there is no reference?

→Thank you for your comment. SCD medium is commonly used as a medium for *Candida* species; therefore, to unify the experimental conditions in this study, SCD medium was used. Sufficient growth was observed in SCD medium for all fungi and bacterium used herein.

At the crystal violet assays, it is not clear how to induce the biofilm formation, so authors need to explain more clearly.

→We have included information on biofilm formation in the Material and methods in lines 154–158.

At the “Germ tube test”, it is not clear the media is appropriate to induce the germ tube. Please check again.

→Thank you for your advice. We have checked our data again, and since significant formation of germ tubes was observed in *C. albicans* cultured in SCD broth, we believe that the medium used in this study is suitable for formation of germ tubes.

At line 166, OD may not correct in there. So, check and change.

→We have double-checked the OD values and added/corrected these in lines 202–204.

Authors frequently use bacteria even though the assay include fungi. So, authors need to change that.

→Thank you for your comment. We have carefully corrected this in the entire manuscript by distinguishing between fungi and bacterium.

At results, authors need to explain the results with the number to make easy to understand.

→We added a description of the results numerically in lines 181–187 and 214–216.

At discussion, Turbidity is not directly indicative for the level of proliferation. So, authors need to change that.

→Thank you for your comment. We have presented turbidity to simply refer to "possibility of proliferation" in lines 148–151.

At discussion, discussion is written usually for explain the results, so if authors add more possible mechanism of HO, that should be a better discussion.

→Thank you for your advice. We mentioned the possible mechanism of HO in the Discussion in lines 288–294.

If the HO is a gel-type, how authors weighing the HO? because weight of HO just includes hinokitiol and IPMP, or include hinokitiol and IPMP with some others?

→Thank you for your question. HO in the tube was placed directly into a sterile 15 mL or 50 mL centrifuge tube and weighed using an electronic balance. In addition to hinokitiol and IPMP, HO contains an anti-inflammatory ingredient (glycyrrhizinate dipotassium), a solvent (purified water), a humectant system (concentrated glycerin, propylene glycol, hydrolyzed collagen, sodium hyaluronate), a solubilizer (polyoxyethylene hydrogenated castor oil), a viscosity adjuster (carboxyvinyl polymer, sodium polyacrylate), a pH adjuster (potassium hydroxide), a preservative (sodium benzoate), a flavoring agent (flavor), and a stabilizer (ethylenediaminetetraacetic acid disodium).

Why authors mixed the hinokitiol and IPMP and what is the ratio?

→Hinola^®^ (HO) is an oral care gel developed by Otsuka Pharmaceutical Co., Ltd. Hinokitiol, which is extracted from *Cupressaceae* plants. It has long been used in daily life for its insect repellent, deodorant, and antibacterial effects. IPMP has broad-spectrum bactericidal properties and is effective against bacteria or fungi. For example, it is often used in acne care products to suppress acne-causing bacteria, and in antiperspirants to suppress the growth of odor-causing bacteria. These two ingredients are still used in various common daily products today, and have been applied in the dental field, leading to the development and commercialization of Hinola^®^ (0.1% hinokitiol and 0.02% IPMP).

At figure 1, the figure captured were compared for each bacterium correctly? I think authors need to double check that.

→Thank you for pointing this out. The acquisition and measurement of the images in figure 1 were all performed under the same conditions. We also reconfirmed that the images captured were correctly compared for each bacterium.

At figure 4, authors need to provide that results statistically and those are hyphae or pseudohyphae? I suggested that authors need to explain about that.

→Thank you for your suggestion. We compared the number of germ tube-producing cells in 500 cells between the control group and the HO-added group, and performed this in three sections. We next statistically analyzed and graphed the results (Fig. 4B).

*Candida albicans* cultured in SCD broth formed biofilms (Fig. 3). Since hyphae are necessary for biofilm formation, we believe that the images are hyphae rather than pseudohyphae (Fig. 4A). We mentioned this in lines 271–272 of the Discussion.

Finally, I could not find table.

→Please accept our sincerest apologies. We have uploaded the missing table.
