# Supplementary material for: Antimicrobial effect of oral care gel containing hinokitiol and 4-isopropyl-3-methylphenol against intraoral pathogenic microorganisms
Source: PLoS One. 2023 Sep 1;18(9):e0283295. doi: 10.1371/journal.pone.0283295 (PMC10473516; doi:10.1371/journal.pone.0283295)
Supplement: Attachment — Submitted filename: Response_to_Reviewers.docx [file pone.0283295.s002.docx]

**Response to Reviewers**

We sincerely appreciate to the reviewers for their supports that have contributed to the improvement of our research paper. We have provided the responses to the comments below:

**Reviewer #2:** There is a table 1 in line 192 and 236, but I could not find table1.

Authors need to increase the resolution of all figure.

→Please accept our sincerest apologies. We have included table 1 as part of our main manuscript after “References”. In addition, we have tried to improve the resolution of the all figures as much as possible. We kindly ask for your confirmation.
